# Resident-generated versus instructor-generated cases in ethics and professionalism training

**DOI:** 10.1186/1747-5341-1-10

**Published:** 2006-06-29

**Authors:** Alexander A Kon

**Affiliations:** 1 Department of Pediatrics and the Program in Bioethics at the University of California Davis, Sacramento, CA, USA

## Abstract

**Background:**

The emphasis on ethics and professionalism in medical education continues to increase. Indeed, in the United States the ACGME will require residency programs to include professionalism training in all curricula by 2007. Most courses focus on cases generated by the course instructors rather than on cases generated by the trainees. To date, however, there has been no assessment of the utility of these two case discussion formats. In order to determine the utility of instructor-generated cases (IGCs) versus resident-generated cases (RGCs) in ethics and professionalism training, the author developed an innovative course that included both case formats. The IGCs were landmark cases and cases from the experience of the course instructors, while the RGCs were selected by the residents themselves. Residents were then surveyed to assess the strengths and weaknesses of each format.

**Results:**

Of twenty-two second and third year residents, fourteen completed surveys (response rate 64%). Residents were nearly evenly split in preferring RGCs (38%), IGCs (31%), or not preferring one to the other (31%). 29% stated that they learn more from the RGCs, 21% stated that they learn more from the IGCs, and 50% stated that they did not find a difference in their learning based on format. In general, residents surveyed prefer a mix of formats. Residents tended to find the RGCs more relevant and interesting, and felt the IGCs were necessary to ensure adequate breadth of cases and concepts.

**Conclusion:**

Based on our relatively small sample at a single institution, we believe that educators should consider incorporating both instructor-generated and resident-generated cases in their ethics and professionalism curricula, and should evaluate the utility of such a model at their own institution. Further work is needed to illuminate other potential improvements in ethics and professionalism education.

## Background

Ethics and professionalism training is becoming ubiquitous in both undergraduate and graduate medical education. Indeed, in the United States the Accreditation Council for Graduate Medical Education (ACGME) now requires that all residency training programs focus resident education around six core competencies, which include interpersonal and communication skills as well as professionalism[1] Designing curricula to address medical ethics and professionalism is challenging. Recently, the American Society for Bioethics and Humanities (ASBH) created a Task Force on Graduate Medical Education on Bioethics and Humanities to develop guidelines for curricula that address the ethical aspects of many of these competencies[2] Experts in the field support case-based learning, however nearly all published curricula that include case-based sessions focus on cases generated by the instructors (i.e., landmark cases and cases from the experience of the educators) [3-7] Few published curricula focus on cases derived from the experience of the trainees, and no data exist establishing the relative benefits of instructor-generated and resident-generated cases for discussion.

There are significant benefits to each method of case presentation. On one hand, instructor-generated cases (IGCs) are significantly easier to facilitate and are less time intensive to prepare. Instructors can develop a portfolio of cases that illustrate major teaching points, and draw upon these predetermined cases in an orderly fashion to build on previous lessons. Since the instructors use the same cases over years, they can have a clear idea of how to facilitate the discussion to best illuminate the salient points.

On the other hand, because resident-generated cases (RGCs) center on patients with whom residents are personally involved, residents may find these discussions more interesting. Optimizing resident interest is important because engaging residents in professionalism training seems, from the author's experience, to be more difficult than in other areas of medical education. For example, in cardiac physiology lectures, residents are taught "right" and "wrong" answers, and they understand that knowing the "right" answer might help them save a patient's life one day. In contrast, when learning about ethics and professionalism, residents must develop the ability to reason through ethical problems when there is often no clear "right" answer. In the author's experience, it is more difficult for residents to see direct benefits to themselves or to their patients through the acquisition of these skills when compared to disease-based lectures. Centering on cases about which the residents have personal experience may help to facilitate instruction by placing the concepts in a more meaningful context.

At the University of California Davis, we developed an innovative curriculum to teach pediatric residents professionalism and ethics in practice. Our curriculum includes an initial lecture series followed by case-based discussions using both IGCs and RGCs. Our course was designated as an exemplar by the ASBH Task Force on Graduate Medical Education on Bioethics and Humanities, and the syllabus is published on their website[8] This course has also been recognized by the ACGME, and is posted on their website as an example for other institutions[9]

Because there is no formal data to suggest that one case format is superior to the other, we hypothesized that a mix of case formats would be superior than a course focusing on either format alone. Ideally, we would have randomly assigned residents to three courses: RGC alone, IGC alone, and RGC/IGC mix. We would then perform pre and post course evaluations of residents' abilities to reason through ethical problems in cases with which they themselves were involved. Such a project was not practical, and there remains substantial debate as to how best assess residents' actual abilities in ethical reasoning. Therefore, we determined that an assessment of residents' opinions of our course and the case formats presented could serve as a beginning for such inquiry. We therefore chose to focus on resident perceptions in three key areas: Do the case discussions cover practical issues that the residents believe they do/will face in clinical practice? Are the case discussions broad enough to cover the wide array of ethical issue they do/will face? And which case format in general do they prefer, which format do they believe facilitates their own learning, and ideally what mix of formats would they prefer? These specific areas were chosen because our education team believed that the differences in case formats would most likely impact these areas. The purpose of this paper is to describe our curriculum and report the findings of our resident survey. These findings may assist other residency programs as they develop ethics and professionalism curricula.

## Methods

The University of California Davis Pediatric Residency Training Program is a fully accredited, three-year program including eleven residents per class. In the Fall of 2000, we developed an innovative curriculum to teach pediatric residents professionalism and ethics in practice. The course was implemented in the summer of 2001, and all residents have participated in the course since its inception.

Our curriculum begins each summer with a five-part lecture series that covers general topics in pediatric ethics as follows: 1) Basic principles of ethics and professionalism; 2) Bringing ethical theory to the bedside; 3) The law and ethics; 4) Delivering bad news; and 5) Ethics of consent and California law regarding minors with the legal authority to consent.

Following the introductory lecture series, monthly case conferences are held at noontime. For these case conferences, we have integrated both IGCs and RGCs. IGCs are landmark cases in pediatric medicine as well as cases from the experience of the course instructor (Kon). These are cases that the instructor has used in ethics education for several years. The RGCs are chosen and presented by the residents themselves. In general, these are ongoing cases from the ward, the neonatal or pediatric intensive care units, or the outpatient clinic. Approximately one week prior to any session designated for an RGC, the course instructor works with the chief and senior residents to find an appropriate case. The intern involved in the case is asked to give a brief presentation at the meeting. We have also incorporated roll-playing and debate formats into some sessions.

After two years of course instruction, all second and third year resident (n = 22) were surveyed to assess their perception of the course. These residents had participated in the ethics and professionalism course since their intern year, and had each attended case discussions using both the IGC and RGC format. Surveys were anonymous, and residents were encouraged to add their comments to the survey forms. Residents were asked to score their responses to statements on a five-point Likert scale (strongly disagree to strongly agree). For analysis, we dichotomized responses to each survey question to agree (score of 4 or 5), or not agree (1 through 3).

Residents were asked which format, IGC or RGC, they preferred, and why. They were asked if they learned more from the IGC or RGC discussions, and why. They were asked about the relevance and breadth of the IGCs and RGCs. Finally, they were asked what proportion of IGCs and RGCs should be used in future years. The complete survey is available from the author upon request. This research was approved by the University of California Davis IRB.

## Results

**Figure 1:**
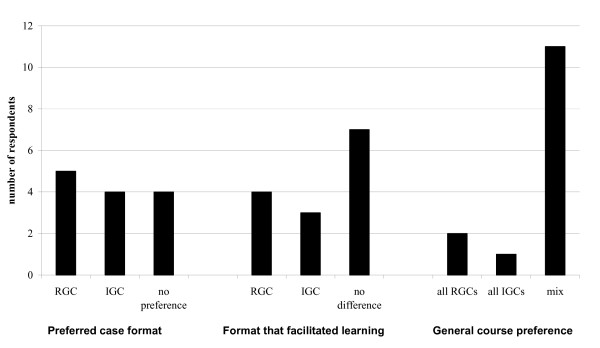
Case format preferences.

**Figure 2:**
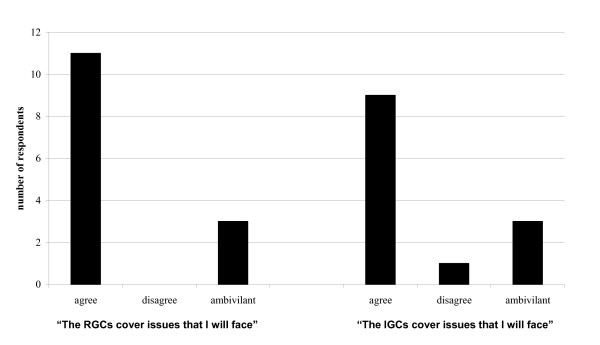
Practicality of issues discussed.

**Figure 3:**
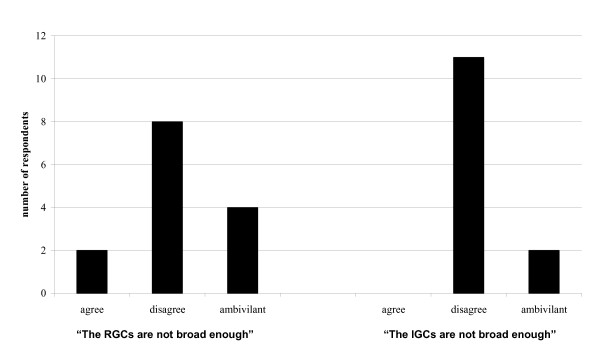
Breadth of cases discussed.

Of the twenty-two second and third year residents, fourteen (64%) completed surveys. Response rates were identical in the two class-years, however some respondents did not answer all questions. Residents were nearly evenly split between preferring IGC, RGC, or both equally and were split in which they believed best facilitated their learning. In general, however, residents felt that a mix of both formats was ideal regardless of which format they personally preferred (Fig 1). Responses to questions regarding how practical residents found sessions and the breadth of cases covered are presented in Figures 2 and 3. When asked what percentage of cases would ideally be IGCs and RGCs, on average, residents wanted 51% of cases to be resident-generated, and 49% to be instructor-generated.

Resident comments in favor of the RGC format included: "(The RGCs) showed me how difficult in practice it is to apply one's ideals." "It helped on the case I was involved in to settle personal issues I had." "(The RGC format is) a better tool to put to immediate use/practice." "I was more interested (in the RGCs)." "(The RGCs) were easier to discuss." "It was easier to see the conflict (in the RGCs)." "(The residents) had a much better grasp of the events surrounding (the RGC) cases."

Other residents also discussed why they prefer the IGC format: "The facilitators usually have more interesting cases to discuss because of more experience." "(There was) more clarity in the issues (in the IGC sessions), and I learned more about 'true ethics'." "Attendings get to the point."

## Discussion

Residents were divided in their preferences for session format. Written comments suggested that they may feel more personally vested in the RGCs and appeared to value the ability to immediately put into practice what they learned. Some residents felt that the RGCs lacked adequate breadth. However, this was not the case with the IGCs. Our interpretation of this data is that curricula in ethics and professionalism should include a mix of both IGCs and RGCs in order to provide residents the opportunity to discuss "real life" cases and to ensure adequate breadth of topics covered.

Despite division regarding which format residents preferred, nearly all stated that they would want a mix of formats in their course. We interpret this finding to suggest that the residents were aware that each case format served a slightly different purpose, and that both purposes were important to their own education. While some residents seemed to prefer discussing cases that were ongoing and others preferred discussing historical cases that were often more nuanced or groundbreaking, we believe that nearly all residents found both aspects important as demonstrated by their overwhelming preference for a course integrating both formats. Further, because some residents preferred the RGC format while others preferred the IGC format, a course presenting both formats can present all residents with optimal learning opportunities during different sessions.

The inclusion of both IGCs and RGCs may be integrated into already existing curricula. Several other training programs have developed ethics and professionalism courses with rich portfolios of instructional cases, and they have reported their positive experiences using them [4-7] Our findings suggest that integration of RGCs in such course may increase resident enthusiasm for, and participation in, ethics and professionalism training.

There are, however, limitations to our findings. Our population was limited to a relatively small number of pediatric residents at one institution. Given perceived differences in attitudes among trainees in different fields of medicine, and at different institutions, it may be that our findings would have been different in a different cohort (e.g., a surgical residency program). Further, because our survey was conducted at the beginning of the academic year, we were unable to include our intern class. It is possible that physicians in their first year of training might have a different perception that those who are more senior, and we were unable to assess such potential differences. Furthermore, with a response rate of only 64%, there may have been a bias in our findings, however it is unlikely that case format preference would influence rate of response, and therefore this should not have significantly affected our findings. Given these limitations, educators may wish to consider incorporating the IGC/RCG format and assessing the efficacy at their own institution. A broader inquiry of the utility of such a format in multiple venues would be of significant value.

Although residents believed that there were benefits to each case format, and generally responded that a course including both formats would be most preferable, this represents the opinion of the residents and may not correlate with the actual utility of the discussion formats. Ideally, a study of how to best design ethics and professionalism courses would test residents' actual behavior in their clinical care, their ability to recognize ethical dilemmas and reason through them, and the lasting effects of training after the residents have left the institution. Such testing was not practical, and indeed there is no well-established method for performing such evaluations. Therefore, we were unable to perform such an inquiry.

It is reasonable to assume, however, that the residents would be better judges of how practical the sessions were, and whether the discussions impacted the care they provide, than the instructors. We focused not only on which case formats they generally preferred, but further asked which format they believed facilitated their own learning. While such a study is not ideal, we believe that it is reasonable to accept that individuals who have successfully completed medical education have good insights into what educational approaches facilitate their own learning. Clearly, however, we were unable to assess whether our course actually improved the care that our residents provide to patients, whether the course improved their ability to recognize ethical dilemmas, and whether they were better able to handle ethically problematic situations in appropriate ways, which ultimately is the goal of any such course.

We believe that for a system that includes discussions of ongoing resident cases to function well, it is paramount that facilitators create an atmosphere wherein residents feel that they can discuss their thoughts and feelings regarding a case without negative ramifications. Creating such a non-judgmental environment may be difficult. However, without such assurances, residents are unlikely to openly discuss possible mistakes and disagreements they have with more senior members of the care team. Further, ongoing evaluation of the quality of such educational programs should be viewed as carrying the same importance as our evaluation other aspects of clinical competence for our physicians-in-training.

## Conclusion

We conclude that educators should consider integrating a mix of resident-generated and instructor-generated cases in their training programs to teach professionalism and ethics. Further investigation should test the possible merits of such a mixed case approach in other venues to supplement this preliminary work. Further, educators ought continue developing and testing other novel approaches to resident education. As the ultimate goal of any such educational program is to produce practitioners who are sensitive to their patients' needs, can communicate effectively with those they care for as well as their colleagues, are able to recognize ethical issues in practice, and embody ethical integrity, we must develop tools to test the true success of our educational endeavors.

## Abbreviations

IGC: Instructor-Generated Cases

RGC: Resident-Generated Cases

## Competing interests

The author(s) declare that they have no competing interests.

## Authors' contributions

AK was responsible for all aspects of this project including curriculum development, course instruction, development of the assessment tool, conduction of resident survey, data interpretation, and manuscript authorship.
